# Assessing Genomic Diversity and Selective Pressures in Bohai Black Cattle Using Whole-Genome Sequencing Data

**DOI:** 10.3390/ani12050665

**Published:** 2022-03-07

**Authors:** Xiaohui Ma, Haijian Cheng, Yangkai Liu, Luyang Sun, Ningbo Chen, Fugui Jiang, Wei You, Zhangang Yang, Baoheng Zhang, Enliang Song, Chuzhao Lei

**Affiliations:** 1 Institute of Animal Science and Veterinary Medicine, Shandong Academy of Agricultural Sciences, Shandong Key Lab of Animal Disease Control and Breeding, Jinan 250100, China; maxiaohui152@163.com (X.M.); 98061107@163.com (H.C.); fgjiang2017@163.com (F.J.); uv790402@163.com (W.Y.); 2 College of Animal Science and Technology, Northwest A&F University, Xianyang 712100, China; nwafuliuyangkai@126.com (Y.L.); sunluyang8869@nwafu.edu.cn (L.S.); ningboch@126.com (N.C.); 3 HuaXing Bohai Black Cattle Co., Ltd., Binzhou 256600, China; wdxhxxm@126.com; 4 Wudi Animal Husbandry and Veterinary Service Management Center of Binzhou City, Binzhou 256600, China; wdslb777@163.com

**Keywords:** Bohai Black cattle, Chinese cattle, whole-genome resequencing, SNPs, genomic diversity, population structure, selection signatures, meat quality, coat color

## Abstract

**Simple Summary:**

Bohai Black cattle are one of the indigenous black coat cattle breeds in China, which are famous for their excellent meat quality. Whole-genome sequencing technology has been extensively developed to study species genome genetic diversity, population structure, selection pressure, demographic events, etc. However, a limited number of studies have reported genomic diversity and selection pressures in Bohai Black cattle. The purpose of this study is to analyze population structure and genomic differences between Bohai Black cattle and five “core” cattle populations from all over the world, mainly oriented on the identification of selection signatures using whole-genome sequencing data. In addition, we identify a series of candidate genes that can potentially be related to black coat color, meat quality, immunity, and reproduction in this breed. This study provides valuable genomic resources and theoretical basis for the future breeding of Bohai Black cattle.

**Abstract:**

Bohai Black cattle are one of the well-known cattle breeds with black coat color in China, which are cultivated for beef. However, no study has conducted a comprehensive analysis of genomic diversity and selective pressures in Bohai Black cattle. Here, we performed a comprehensive analysis of genomic variation in 10 Bohai Black cattle (five newly sequenced and five published) and the published whole-genome sequencing (WGS) data of 50 cattle representing five “core” cattle populations. The population structure analysis revealed that Bohai Black cattle harbored the ancestry with European taurine, Northeast Asian taurine, and Chinese indicine. The Bohai Black cattle demonstrated relatively high genomic diversity from the other cattle breeds, as indicated by the nucleotide diversity (pi), the expected heterozygosity (H_E_) and the observed heterozygosity (H_O_), the linkage disequilibrium (LD) decay, and runs of homozygosity (ROH). We identified 65 genes containing more than five non-synonymous SNPs (nsSNPs), and an enrichment analysis revealed the “ECM-receptor interaction” pathways associated with meat quality in Bohai Black cattle. Five methods (CLR, θπ, F_ST_, θπ ratio, and XP-EHH) were used to find several pathways and genes carried selection signatures in Bohai Black cattle, including black coat color (*MC1R*), muscle development (*ITGA9*, *ENAH*, *CAPG*, *ABI2*, and *ISLR*), fat deposition (*TBC1D1*, *CYB5R4*, *TUSC3*, and *EPS8*), reproduction traits (*SPIRE2*, *KHDRBS2*, and *FANCA*), and immune system response (*CD84*, *SLAMF1*, *SLAMF6*, and *CDK10*). Taken together, our results provide a valuable resource for characterizing the uniqueness of Bohai Black cattle.

## 1. Introduction

Abundant cattle resources are essential for the diversified development of animal husbandry in China and are an important constituent form of agricultural economy. Cattle have established a close relationship with human beings in economic and cultural aspects by providing most of the leather, meat, and milk [1]. Since cattle domestication [2], a wide range of natural and artificial selection events in cattle have greatly changed its customary behavior and appearance [3,4]. The various cattle breeds still show considerable diversity in terms of milk and meat production, fertility, coat color, body size, horns, etc. [5,6,7]. With the latest development of national breeding programs, there is a need to conduct genetic research on important phenotypic traits and economic traits (such as coat color and meat quality) of cattle breed.

**Figure 1:**
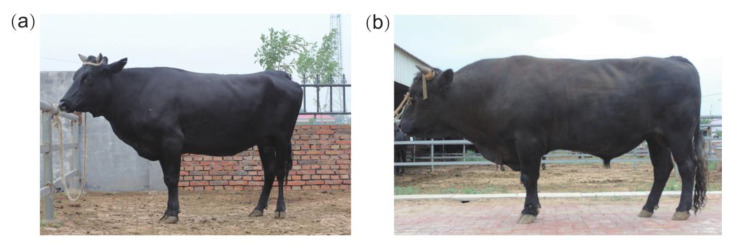
Pictures of Bohai Black cattle: (**a**) female; (**b**) male.

As one of rare black breeds, Bohai Black cattle is recognized for its unique black coat, hooves, horns, nose, and tongue (Figure 1) [8]. In the early period, Bohai Black cattle were mainly used as a farming tool in order to facilitate agricultural production practice [9]. However, since the 2000s, with the popularization of agricultural mechanization, the advantages of Bohai Black cattle in agricultural production have gradually fade, and the number of Bohai Black cattle has dropped sharply (50,000 to 20,000) in the past 20 years [10]. Currently, a national breeding program has improved Bohai Black’s meat quality, particular in fat-marble deposition trait, which is in line with Asian people’s pursuit of beef [9].

Previous studies have explored the genetic diversity and origin evolution of Bohai Black cattle using microsatellite, as well as mitochondrial and Y-chromosome markers [11,12,13]. These findings have suggested that Bohai Black cattle were of mixed origin of taurine and indicine cattle and had high genetic diversity [11,12,13]. Earlier studies on coat color and meat quality traits in Bohai Black cattle have been mainly conducted through candidate gene scan [14,15]. Analysis of the *MC1R* gene by PCR amplification and DNA sequencing has revealed its association with coat color in cattle and that the allele ED and E+ are associated with the synthesis of the black pigment. Analogously, the bovine bone morphogenetic protein 15 (*BMP 15*) gene of Bohai Black cattle was genotyped by PCR-SSCP and found to be associated with meat quality traits [14,15].

WGS has become an effective method to detect population structure and to identify genomic selection signatures in cattle [16]. In 2018, a prominent study revealed three distinct ancestries of East Asian cattle populations, i.e., East Asian taurine, Eurasian taurine, and Chinese indicine, by using WGS data from five “core” cattle populations (European taurine, Eurasian taurine, East Asian taurine, Chinese indicine, and South Asian indicine) from around the world [17]. A number of studies have focused on the European *Bos Taurus* cattle [18], *Bos indicus* cattle [19], African taurine cattle [20], and the adaptable indigenous cattle breeds [21]. However, there have been fewer reports on the genomic variation of Bohai Black cattle. Here, we performed the population structure, genomic diversity, and selection pressures in Bohai Black cattle by using the WGS data of 10 Bohai Black cattle and 50 cattle from five “core” cattle populations, which can comprehensively assess genomic variation of Bohai Black cattle.

## 2. Materials and Methods

### 2.1. Sample Collection and Whole-Genome Sequencing

Five ear tissue samples of Bohai Black cattle were collected from the incorporated company of HuaXing Bohai Black Cattle Co., Ltd. of Binzhou Wudi in Shandong and sent to the Novogene Bioinformatics Institute (Beijing, China) (Table S1). The pair-end libraries were constructed for each individual (500 bp insert size) and sequenced. Additionally, published data of Bohai Black (*n* = 5, Table S1), European taurine (Angus (*n* = 9) and Simmental (*n* = 8) cattle); Northeast Asian (Hanwoo cattle (*n* = 10)); Chinese indicine (Ji’ an (*n* = 4), Jinjiang (*n* = 3), and Wenshan (*n* = 6) cattle); and South Asian indicine cattle (Brahman (*n* = 4), Gir (*n* = 2), Nelore (*n* = 1), Sahiwal (*n* = 1), Hariana (*n* = 1), and Tharparkar (*n* = 1) cattle) were also collected (Table S2).

### 2.2. Reads Mapping and SNP Calling

The clean reads of each individual sample were mapped against the *Bos.taurus* reference genome ARS-UCD1.2 using the Burrows–Wheeler Aligner (v0.7.13-r1126) program [22] with default parameters. We used the Genome Analysis Toolkit 3.8 (GATK) for downstream SNP calling [23]. Then, we obtained the high-quality raw SNPs by using the module “VariantFiltration” with the parameters (DP < 249 (1/3-fold total sequence depth for all individuals)||DP > 2245 (three-fold of total sequence depth for all individuals)||QD < 2.0||FS > 60.0||MQ < 40.0||MQ RankSum < −12.5||ReadPosRankSum < −8.0||SOR > 3.0) of GATK. Finally, we used the ANNOVAR software [24] to identify the nsSNPs of Bohai Black cattle. In addition, enrichment analyses of GO and KEGG pathways were performed to evaluate the functional importance for genes containing specific nsSNPs using the web-based platform KOBAS (http://kobas.cbi.pku.edu.cn/kobas3/genelist/, accessed on 9 December 2021) [25]. In addition, the SNP density of each cattle breed was calculated by VCFtools (window 100,000) [26].

### 2.3. Population Genomic Parameter Analysis

In order to reveal the genome genetic diversity of Bohai Black cattle, the nucleotide diversity (pi), expected heterozygosity (He) and observed heterozygosity (Ho), the linkage disequilibrium (LD) decay, and the runs of homozygosity (ROH) of all cattle populations were calculated and compared. We used VCFtools [26] to estimate genomic nucleotide diversity of each breed (--window-pi 50,000 --window-pi-step 20,000), and PLINK v1.9 was used to compute the expected heterozygosity (H_E_) and the observed heterozygosity (H_O_) with default settings [27]. As for the LD decay with physical distance between SNPs, it was calculated by PopLDdecay software [28]. Based on the number of autosomal SNPs, the ROH of each individual was calculated by PLINK (--homozyg-window-snp 50). We primarily calculated the total number of ROHs (0.5–1 Mb, 1–2 Mb, 2–4 Mb, and >4 Mb) per breed and the length of ROHs for all individuals per breed.

### 2.4. Population Genetic Structure and Phylogenetic Analysis

The Vcftools v0.1.12 (https://vcftools.github.io/index.html, accessed on 9 December 2021) software [26] converted the vcf files into plink format. The PLINK (version 1.9) software [27] was used to remove the linkage sites in the genomic data with parameters of -indep-pair-wise 50 5 0.2 option of PLINK (version 1.9), and the filtered data were used for the analyses of principal component analysis (PCA), the phylogenetic tree, and the ADMIXTURE analysis. We used the ADMIXTURE software [29] to estimate the ancestral composition of each individual using genome-wide unlinked sites. The study analyzed the possible ancestral origins of 60 individuals (K = 2 to K = 4). The results were visualized via R 3.6.1 software. The PCA of 60 individuals was performed by the smartPCA of the EIGENSOFT v5.0 package [30] to estimate the eigenvectors. The Tracy–Widom distribution was used to assess the significance of each principal component, and the results of the first and second principal components were plotted using the ggplot2 package in the R 3.6.1 software. The model of neighbor-joining (NJ) tree used for phylogenetic reconstruction was constructed based on the pairwise genetic distances matrix supplied by PLINK using MEGA v7.0 [31], and was visualized using iTOL (https://itol.embl.de/, accessed on 9 December 2021) [32].

### 2.5. Genomic Signatures of Positive Selection

Based on the statistical results of SNPs and densities, then, we performed the detection of positive selection signals. In this study, the composite likelihood ratio (CLR) and the nucleotide diversity (θπ) methods were used to detect the selection signatures within Bohai Black cattle [33]. The CLR was computed by using the SWEEPFINDER2 software to calculate SNPS within a non-overlapping 50 kb window [34] and θπ was estimated using VCFtools (50 kb sliding window and 20 kb step) [26]. Consistent with our previous methods, the top 1% window was selected as the candidate region under selection [17].

We also used the fixation index (*F_ST_*), the nucleotide diversity analysis (θπ ratio), and XP-EHH methods to identify the potential areas differentially [35] between Bohai Black and Brahman cattle. The fixation index (*F_ST_*) values and the high differences in genetic diversity (θπ ratio) were calculated in 50 kb sliding windows with 20 kb steps along the autosomes using VCFtools [26]. For the XP-EHH method, our test statistic was the average normalized XP-EHH score in each 50 kb region. Similarly, the top 1% regions (*F_ST_* top 1%, θπ ratio top 1%, and XP-EHH top 1%) were selected as candidates for positive selection in these three methods. Of course, we finally selected the overlapping regions of two or three methods as candidate regions in order to make our results more reliable. We also used VCFtools to calculate the Tajima’s D statistic within a small range for the candidate genes. Finally, the selected candidate regions were annotated into the reference genome (ARS-UCD1.2), and the candidate genes were analyzed for functional enrichment of KEGG and GO pathways by using the KOBAS (http://kobas.cbi.pku.edu.cn/kobas3/genelist/, accessed on 9 December 2021) [25]. The KEGG and GO pathways were considered to be significantly enriched only when the *p*-values were less than 0.05.

## 3. Results

### 3.1. Sequencing and SNPs Calling

The average sequencing depth of the reads in individual genomes of 10 Bohai Black cattle and the 50 cattle from five “core” cattle populations were 10.14× and 12.94×, respectively (Tables S1 and S2). In total, ~0.6 billion filtered reads were generated with an average alignment rate of 98.52% in Bohai Black cattle. In addition, a total of 25,982,011 SNPs were detected in Bohai Black cattle (Table S3), of which 1,863,158 SNPs were specific to Bohai black cattle (Figure S1a). The SNPs distribution of all cattle populations in different genomic regions are showed in Table S3. The SNPs included untranslated region (UTR) variant, downstream and upstream variant, intergenic, intron, exonic splice variant, stop gain, and stop loss variant. In terms of the numbers of SNPs, the highest number of SNPs was found in Chinese indicine (38,561,826), followed by crossbred Bohai Black (25,982,011), Asian indicine (25,259,144), Hanwoo cattle (12,833,552), and Simmental cattle (10,563,224) (Table S3). The Angus showed the lowest number of SNPs. We also detected 252,638 INDELs that were specific to Bohai Black cattle (Figure S1b). In addition, a total of 506,995,560 transitions (Ts) and 214,567,924 transversions (Tv) were observed in all of the SNPs. The statistical results of SNP density on each chromosome of each cattle breed are shown in Table S4. The SNP density of Bohai Black cattle was 10 variants/KB (Table S4). According to the methods already reported [36], we obtained 7438 specific nsSNPs in Bohai Black cattle by using the ANNOVAR software [24]. We selected genes containing > 5 nsSNPs for Bohai Black and a total of 65 genes were identified in Bohai Black. These genes were significantly associated (*p*-value < 0.05) to only one KEGG pathway and 3 GO terms (Table S5). We found the most significant KEGG pathway was “ECM-receptor interaction”.

### 3.2. Population Genetic Structure and Phylogenetic Analysis

**Figure 2:**
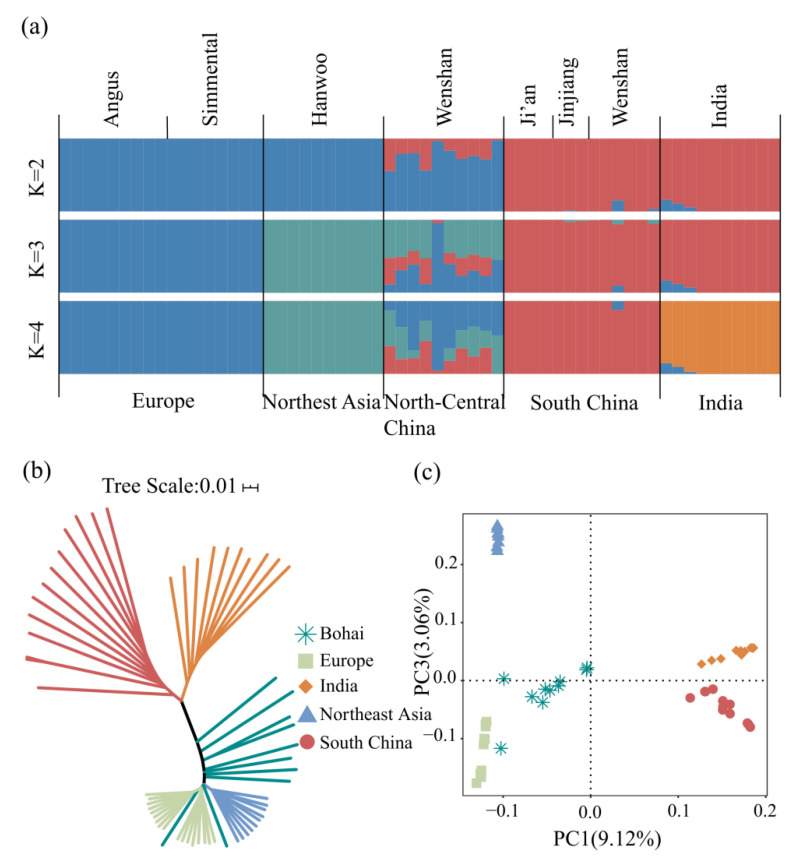
Population structure and relationships of Bohai as compared with several possible ancestral breeds: (**a**) Model-based clustering of cattle breeds using ADMIXTURE with K = 2 to K = 4. Breeds are colored by geographic regions and labeled with breed names; (**b**) neighbor-joining tree of the relationships between the cattle breeds (60 individuals); (**c**) PCA clustering.

To explore the relationship among Bohai Black cattle and five “core” cattle populations, we examined the patterns of genetic differentiation and genomic structure using structure analysis, neighbor-joining (NJ) tree analysis, and principal component analysis (PCA) (Figure 2).

The ADMIXTURE results of a total of 60 individuals showed that K = 2 only separated *Bos indicus* and *Bos taurus*. At K = 4, Bohai Black cattle shared genome ancestrally with European taurine (47%), Northeast Asian taurine (29%), and Chinese indicine (24%) (Figure 2a). The results indicated that Bohai Black cattle was a hybrid dominated by taurine cattle ancestry (76%). PC1 and PC3, respectively, explaining 9.12% and 3.06% of the total variations, separated *Bos indicus* and *Bos taurus*, and Chinese indicine and South Asian indicine, respectively (Figure 2c). The NJ tree showed that all taurine cattle (European taurine and Northeast Asian taurine cattle) and all indicus cattle (Chinese indicine and South Asian indicine cattle) were grouped together, and Bohai Black cattle were located on the side closed to the *Bos taurus*. However, there were two Bohai Black cattle individuals grouped with European taurine cattle. Similar to NJ tree analysis, PCA clustering also revealed clear breed structures (Figure 2b).

### 3.3. Population Genomic Parameter Analysis

**Figure 3:**
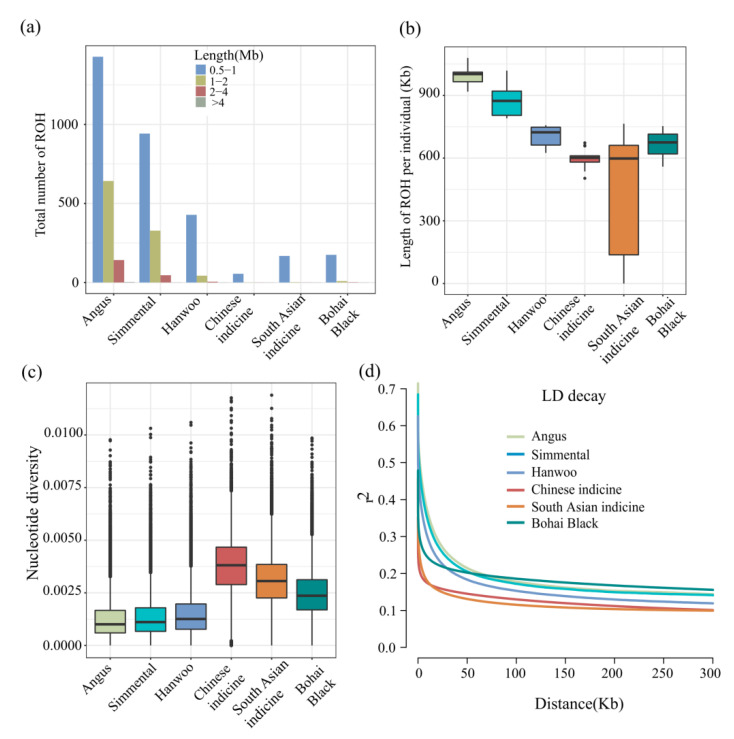
Summary statistics for genomic variation: (**a**) The distribution of total number of ROH across chromosomes; (**b**) the distribution of the lengths of ROH in each breed; (**c**) genome-wide distribution of nucleotide diversity of each breed in 50 kb windows with 20 kb steps (the horizontal line inside the box indicates the median of this distribution, box limits indicate the first and the thirds quartiles, points show outliers); (**d**) genome-wide average LD decay estimated from each breed.

We found that the most of the ROH that were identified in all cattle breeds were between 0.5–1 Mb in length. The medium (1–2 Mb) and long ROH (2–4 Mb) were identified in European taurine cattle (Angus and Simmental cattle) (Figure 3a and Table S6). As compared with the five “core” cattle populations, the total length of the ROH in Bohai Black cattle was medium, lower than that of European commercial cattle breeds (Angus and Simmental cattle) (Figure 3b and Table S7). In addition, the pi value in the Bohai Black cattle (0.002459) was high, second to that of Chinese indicine and South Asian indicine (Figure 3c and Table S8), and the lowest pi value was found in taurine cattle (European and Northeast Asian cattle). The H_E_ and the H_O_ value of the Bohai Black cattle ranked second in all cattle breeds (Table S8). Regarding LD decay, it was nearly consistent with those from the ROH profile. The lowest average genome-wide LD was observed in South Asian indicine cattle and Chinese indicine cattle and the highest value of LD was observed in Bohai Black cattle, followed by European taurine cattle (Angus and Simmental) and Hanwoo cattle (Figure 3d).

### 3.4. Genomic Signatures of Positive Selection

**Figure 4:**
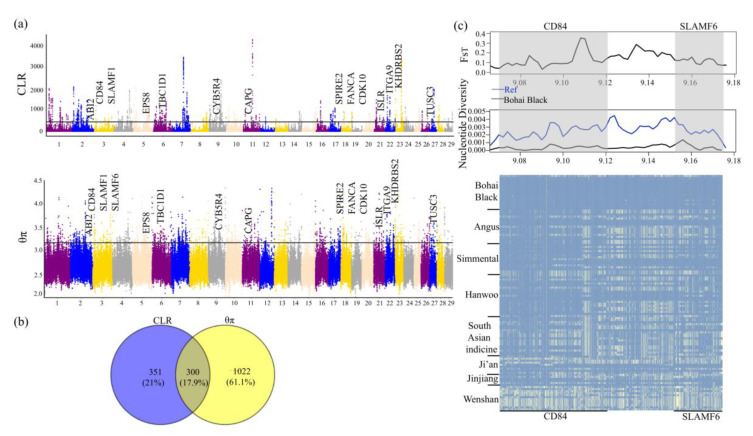
CLR and θπ related results in Bohai Black cattle: (**a**) Manhattan plots showing the results for the autosomes; (**b**) Venn diagrams of genes shared by CLR and θπ methods; (**c**) *F_ST_*, nucleotide diversity, and degree of haplotype sharing across populations at the *CD84* and *SLAMF6* genes. The major allele at each SNP position is colored in yellow.

We performed the CLR and θπ methods to screen for the potential region under selection in the genome of Bohai Black cattle. A total of 671 (CLR) and 1374 (θπ) genes in Bohai Black cattle were identified (Tables S9–S11), of which 300 were overlapped; some genes can be considered as potential candidates for positive selection using CLR and θπ methods in Bohai Black cattle (Figure 4a). These candidate genes were enriched in KEGG and GO pathways in order to more fully explain their potential functions. The enriched KEGG pathway of “ubiquitin mediated proteolysis, bta04120” and “regulation of actin cytoskeleton, bta04810” involving four genes (*ABI2*, *ENAH*, *CAPG*, and *PIK3CA*) that might also be related to muscle development in Bohai Black cattle (Table S8) [37,38] In addition, the GO enrichment analysis revealed several pathways involving important biological processes such as “actin polymerization or depolymerization, GO:0008154 ” (*ABI2*, *ENAH,* and *CAPG*), “ubiquitin protein ligase binding, GO:0031625”, and “UV-damage excision repair, GO:0070914” (*MC1R* and *DDB2*) (Table S12). All of these pathways had *p*-values less than 0.05. In particular, some overlapped candidate genes were strongly selected in Bohai Black cattle and related to muscle development (*ITGA9*, *ENAH*, *CAPG*, *ABI2*, and *ISLR*) [39,40], fat deposition (*TBC1D1*, *CYB5R4*, *TUSC3*, and *EPS8*) [41,42,43], reproduction traits (*SPIRE2*, *KHDRBS2*, and *FANCA*) [44,45], and immune system (*CD84*, *SLAMF6*, *SLAMF1*, and *CDK10*) [46,47,48] in Bohai Black cattle (Tables S9 and S10, Figure 4a). Interestingly, the selection signal was found on BTA3 (3:9070076-9175511) and contained the *CD84* and *SLAMF6* genes from the two tests (Figure 4b,c). These genes also showed a strong signal positive selection in Bohai Black cattle as compared with other cattle breeds from five “core” cattle population (Figure 4c).

**Figure 5:**
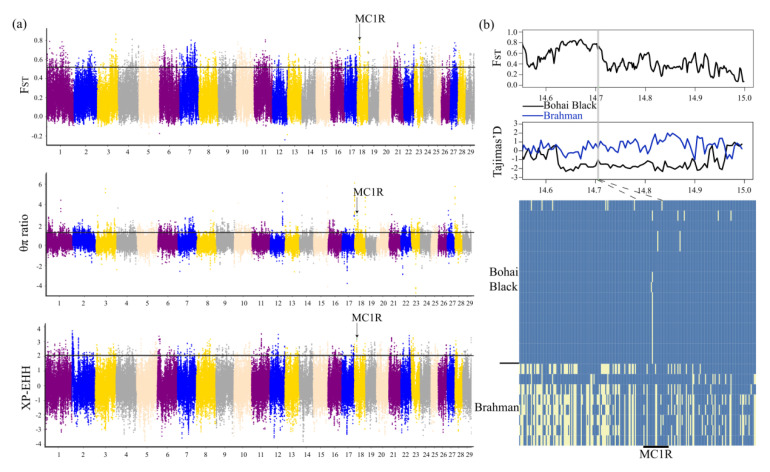
*F_ST_*, θπ ratio, and XP-EHH related results in Bohai Black cattle: (**a**) Manhattan plots showing the results for the autosomes; (**b**) *F_ST_*, Tajimas’ D, and degree of haplotype sharing across populations at the *MC1R* gene region. The major allele at each SNP position is colored in yellow.

Black coat color is the main characteristic of Bohai Black cattle, therefore, we selected Brahman cattle with light coat color to detect the positive selection characteristics in Bohai Black cattle. We also applied the *F_ST_* (*p* < 0.005, *F_ST_* ≥ 0.51959), θπ ratio (*p* < 0.005, θπ ratio ≥ 1.25612), and XP-EHH (*p* < 0.005, XP-EHH ≥ 2.08) methods to detect the positive selection signatures between Bohai Black and Brahman cattle (Tables S13–S15). Sixty-three overlapped candidate genes were scanned by three selection methods (Table S16), and the strongest signal was the *MC1R* gene that associated with melanin deposition (Figure 4a,b). The “melanogenesis, bta04916” and “UV damage excision repair, GO:0070914” pathways containing only the *MC1R* gene were both found by KEGG and GO functional enrichment analysis (Table S17). It is worth noting that the *MC1R* gene was detected among the five mentioned selection methods (Tables S11 and S16), indicating that it was strongly selected in Bohai Black cattle and may be related to their black coat color [49]. The *MC1R* gene was the candidate gene selected by *F_ST_*, θπ ratio, and XP-EHH; through the calculation of *F_ST_* and Tajimas’ D with a smaller window, significant differentiation was observed between Bohai Black and Brahman cattle (Figure 5a,b).

## 4. Discussion

The main purpose of this study was to investigate the genomic diversity of Bohai Black cattle from an overall perspective and reveal the selection pressure of Bohai Black cattle using the whole-genome sequences of 10 Bohai black cattle and 50 cattle from five “core” populations. The ancestral contributions of Bohai Black cattle originated from European taurine (0.467), Northeast Asian taurine (0.291), and Chinese indicine (0.243). We found that Bohai Black cattle are crossbred cattle composed of 76% *Bos taurus* ancestry and 24% *Bos indicus* ancestry, which was consistent with the results recently reported by Liu Z et al. [50]. In the NJ tree, two Bohai Black individuals were separated from others and located on the side of European taurine cattle, indicating that their bloodlines were not pure.

Investigating genetic diversity parameters of populations is essential for the development of future breeding goals [51]. In our study, the results of pi, H_E,_ and H_O_ illustrated that Bohai Black cattle had relatively high genomic diversity. This has been demonstrated in both previous microsatellite studies and recent whole gene sequencing analyses [11,50]. In addition, a relatively large amount of 0.5–1 Mb of ROH were detected within the Bohai Black cattle genome, which was consistent with ROH results in other crossbred cattle [52]. The results of the ROH together with LD decay also reflected the rich genomic diversity and the presence of inbreeding in Bohai Black cattle. This is because, in the past 20 years, with agricultural modernization and the blind introduction by humans, the number of purebred Bohai Black cattle has decreased dramatically. In addition, breeding still employs artificial insemination techniques, uses fewer bulls, and greatly increases inbreeding. This result also provided a direction for future breeding strategies for Bohai Black cattle.

The total number of SNPs within the Bohai Black cattle genome was between indicine and taurine cattle, while Angus cattle had the least total number of SNPs. This presentation pattern of SNPs numbers was consistent with those of previous research [17]. We also identified 65 genes with more than five specific nsSNPs > 5 specific nsSNPs of Bohai Black cattle. These genes can be significantly associated (*p*-value < 0.05) to only one “ECM-receptor interaction” KEGG pathway and 3 GO terms. ECM is a complex mixture of structural macromolecules composed of different proteins such as collagen and glycoprotein, which regulates the proliferation and differentiation of cells and plays an important role in the morphogenesis of tissues and organs [53]. Iqbal N et al. also reported the significant enrichment of the ECM-receptor pathway in Pakistan beef cattle [53]. This suggests that the “ECM–receptor interaction” may be related to the meat quality traits of Bohai Black cattle [53].

Infectious diseases such as bovine brucellosis, bovine tuberculosis (bTB), bovine ephemeral fever (BEF), and some diseases caused by low resistance have been the main threats to the survival of cattle [54]. Therefore, natural selection can have a strong impact on innate immune genes in cattle. Bohai Black cattle have a significant fat-marble deposition trait. Bohai Black cattle are known for strong disease resistance, excellent reproductive performance, and excellent meat quality [10]. We found a region on BTA3 (3:9070076-9175511) containing two candidate genes (*CD84* and *SLAMF6*) under strong selection in Bohai Black cattle in two methods (CLR and θπ). The different haplotype patterns of Bohai Black cattle from those of other cattle illustrated that this region is strongly under selection (Figure 5b). Meat quality is a quantitative trait regulated by complex factors such as glycolysis, stress reaction, proteolysis, ubiquitin mediated proteolysis, apoptosis, and regulation, among others [55]. There were 300 overlapped positively selected genes by both CLR and θπ methods, which significantly overrepresented KEGG pathways (“regulation of actin cytoskeleton, bta04810” and “ubiquitin mediated proteolysis, bta04120”) and GO terms associated with muscle development and fat deposition. The *ENAH* gene is a cytoskeleton regulatory protein involved in the regulation of cell motility and adhesion, and demethylation of it induces overexpression of microRNAs during osteogenic differentiation [37]. The *ITGA9* gene has been reported to be a differentially expressed gene of lncRNAs associated with muscle growth and development in Japanese Flounder (*Paralichthys olivaceus*) [39]. The *CAPG* gene, a member of the coagulate protein family, can regulate spine morphogenesis [38]. The *ABI2* gene is involved in regulating actin cytoskeleton reorganization through the production of tyrosine kinases [56]. The *ISLR* (the immunoglobulin superfamily containing leucine-rich repeat) gene can stabilize canonical Wnt signaling and promote skeletal muscle regeneration [40]. These genes could be associated with muscle development in Bohai Black cattle. However, this was just speculation, and more theoretical and experimental supports are needed. In addition, some genes associated with fat deposition and reproduction have also been identified overlapped by both methods. The *TBC1D1* gene contributes to the development of obesity by regulating skeletal muscle insulin sensitivity [57]. The *CYB5R1* gene acts as an electron source for stearoyl-CoA desaturase (SCD) during fatty acid desaturation [58]. It has been reported as a candidate gene related to meat tenderness and oleic acid percentage in Jiaxian Red cattle and Japanese Black cattle [42,59]. These studies provide more evidence that the *CYB5R4* gene may be related to fat-marble deposition trait of Bohai Black cattle. The *KHDRBS2* gene has been found to be possibly associated with reproduction traits under positive selection in goats using a genome-wide association study (GWAS) [44]. The *SPIRE2* and *FANCA* genes were identified as candidate genes related to reproduction in indigenous Chinese pigs [45]. Therefore, we boldly speculate that these genes are associated with reproductive performance in Bohai Black cattle. Our results indicated that these genes are under positive selection which may be associated with long-term beef selection breeding in Bohai Black cattle.

The Bohai Black cattle genome showed signs of selection in the *MC1R* gene in the “melanogenesis” and “UV damage excision repair” critical pathways. For mammals, melanin deposition in hair is the first line of defense against UV damage and melanin can reduce the penetration of UV into the skin and effectively reduce the damage of UV radiation to cells. The melanocortin 1 receptor (*MC1R*) gene belongs to the G protein-coupled receptor family and is considered to be a major regulator of most processes involved in pigment production and distribution and synthesis throughout the skin [60]. Studies have firmly established that the *MC1R* gene is a key player in UVR-induced tanning and DNA repair mechanisms [61,62]. It has been reported that the genetic variation of *MC1R* gene is associated with the pattern of hair coloration in domestic animals such as cattle, pigs, and chickens [63,64,65]. In particular, a recent study identified the *MC1R* gene in endangered Zhoushan cattle associated with their black coat color at the genomic level [63]. Therefore, we speculate that the *MC1R* gene may be associated with melanin deposition in Bohai Black cattle, but its function needs further exploration.

## 5. Conclusions

This study provides a comprehensive overview of genomic diversity and selective pressures of Bohai Black cattle using whole-genome sequencing data. The population structure analysis revealed that Bohai Black cattle harbored ancestries with European taurine, Northeast Asian taurine, and Chinese indicine cattle. This study points to relatively high genomic diversity on the Bohai Black cattle genome. Our investigation identified many putative genomic regions under positive selection in the Bohai Black cattle genome. Some genes were likely associated with black coat color, immune responses, reproduction, and meat quality traits in Bohai Black cattle, reflecting economic trait evolution under different selection goals. Our results provide a theoretical basis for the genetic evaluation and utilization of Bohai Black cattle.

## Data Availability

Sequence data have been deposited in GenBank (the BioProject accession number PRJNA781398).

## Supplementary Materials

The following are available online at https://www.mdpi.com/article/10.3390/ani12050665/s1, Figure S1: Venn diagram showing overlapping and unique SNPs between the different cattle breeds or groups. The numbers in the circle components show specific SNPs for each breed or overlapping SNPs among breeds or groups. (a) The unique and shared SNPs between Bohai Black and “core” cattle groups. (b) The unique and shared InDels between Bohai Black and “core” cattle groups, Table S1: Summary of newly sequenced and published data of Bohai Black cattle, Table S2: List of additional cattle samples for analysis of genetic background in Bohai Black cattle, Table S3: Distribution of SNPs identified in cattle breeds within various genomic regions annotated by ANNOVAR, Table S4: Density statistics of SNP distribution in each chromosome for each cattle breed (Variants/KB), Table S5: KEGG and GO enrichment results for the genes containing specific nsSNPs > 5 in Bohai Black cattle, Table S6: Statistical results of ROH classification for all cattle breeds, Table S7: Descriptive statistical results of ROH for all cattle breeds, Table S8: Statistical results of pi, H_E_, and H_O_ for all cattle breeds, Table S9: A summary of genes from CLR in Bohai Black cattle, Table S10: A summary of genes from θπ in Bohai Black cattle, Table S11: A summary of genes from CLR and θπ in Bohai Black cattle, Table S12: KEGG and GO enrichment results of Bohai Black candidate genes overlapped by CLR and θπ methods, Table S13: A summary of genes from *F_ST_* in Bohai Black cattle (compared to Brahman cattle), Table S14: A summary of genes from θπ ratio in Bohai Black cattle (compared to Brahman cattle), Table S15: A summary of genes from XP-EHH in Bohai Black cattle (compared to Brahman cattle), Table S16: A summary of genes from *F_ST_* θπ ratio and XP-EHH in Bohai Black cattle (compared to Brahman cattle), Table S17: KEGG and GO enrichment results of candidate genes between Bohai Black and Brahman cattle overlapped by *F_ST_*, θπ ratio, XP-EHH methods.
