# Supplementary figures and images for: Assessing Genomic Diversity and Selective Pressures in Bohai Black Cattle Using Whole-Genome Sequencing Data
Source: Animals (Basel). 2022 Mar 7;12(5):665. doi: 10.3390/ani12050665 (PMC8909316; doi:10.3390/ani12050665)

(a)

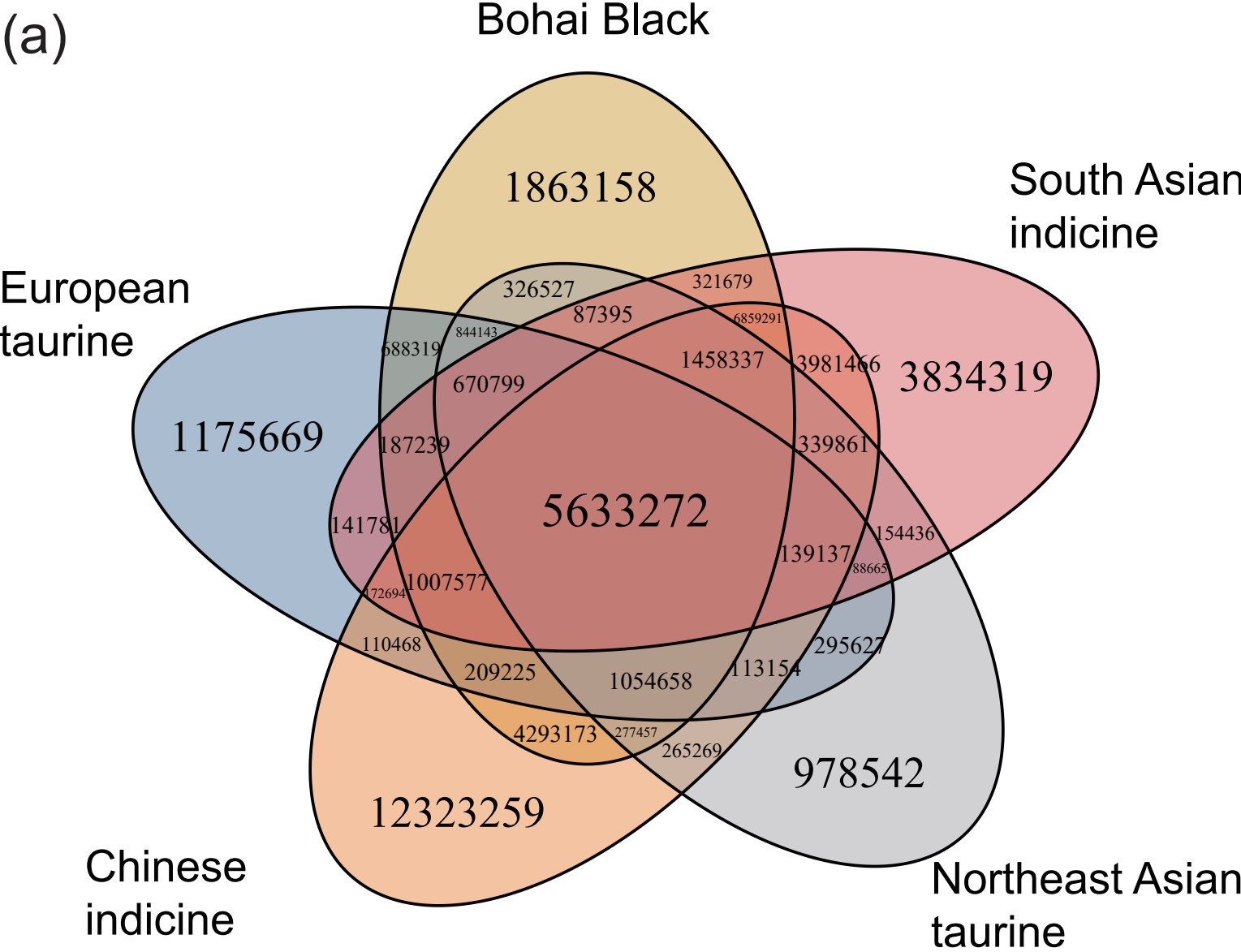

(b)

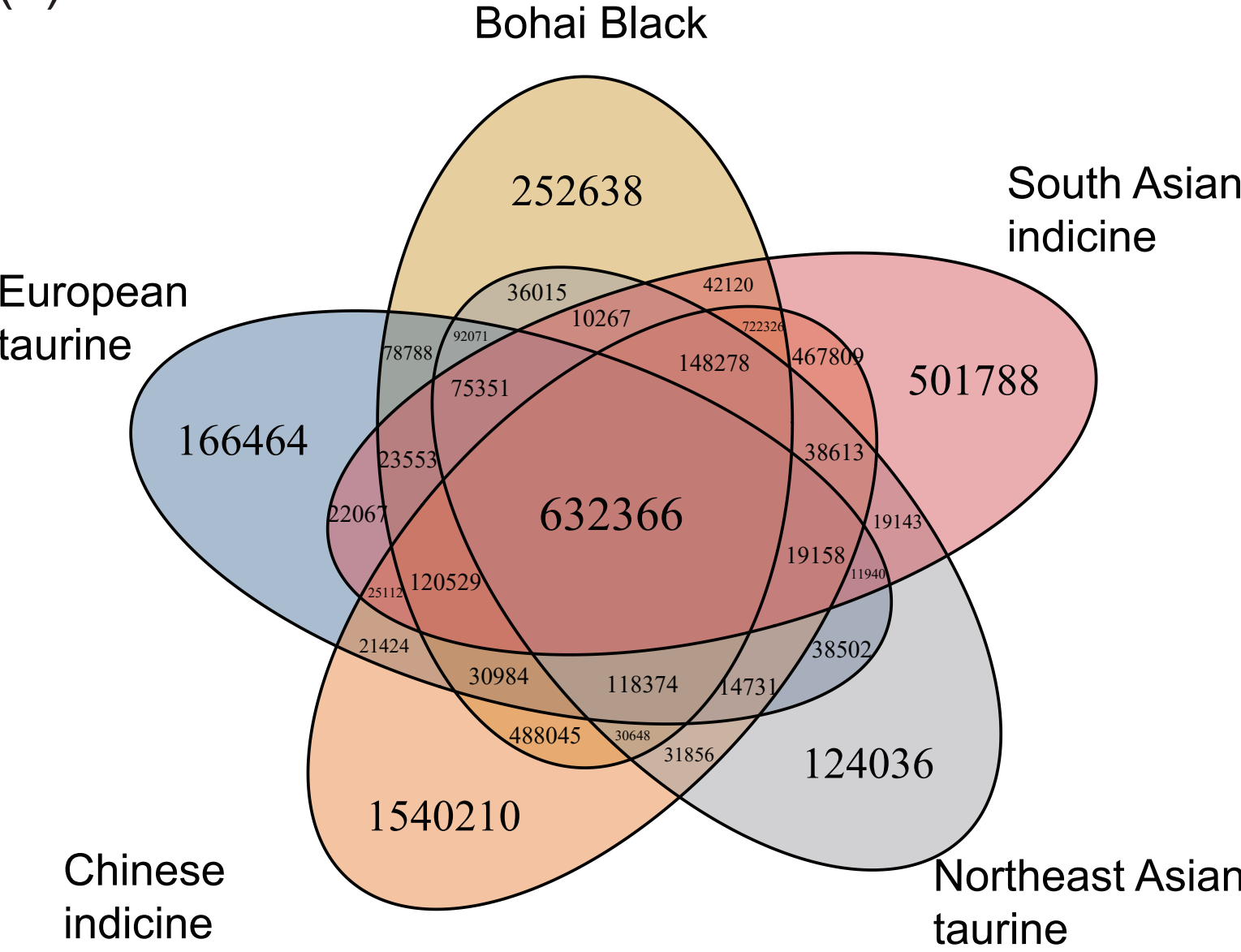

Supplement: Supplementary file 1 [file animals-12-00665-s001.zip › animals-1518598-supplementary/animals-1518598-3.7-supplementary/Figure S1.pdf]
